# The Effects of Laser Acupuncture Therapy on Nocturnal Enuresis: A Systematic Review and Meta-Analysis

**DOI:** 10.1089/acu.2022.0002

**Published:** 2022-08-17

**Authors:** Gil Ton, Chia-Hui Lin, Wen-Chao Ho, Wan-Yu Lai, Hung-Rong Yen, Yu-Chen Lee

**Affiliations:** ^1^ College of Chinese Medicine, Graduate Institute of Acupuncture Science, China Medical University, Taichung, Taiwan.; ^2^ Department of Acupuncture, China Medical University Hospital, Taichung, Taiwan.; ^3^ Department of Public Health, China Medical University, Taichung, Taiwan.; ^4^ Department of Chinese Medicine, China Medical University Hospital, Taichung, Taiwan.; ^5^ College of Chinese Medicine, Graduate Institute of Chinese Medicine, China Medical University, Taichung, Taiwan.; ^6^ Chinese Medicine Research Center, China Medical University, Taichung, Taiwan.

**Keywords:** nocturnal enuresis, low-level laser therapy, laser acupuncture, acupuncture

## Abstract

**Introduction:**

Nocturnal enuresis (NE), often known as bedwetting, is a common condition in children and, as a result, they may have subsequent social impairments. The aim of this study was to evaluate the efficacy of low-level laser therapy (LLLT) in children with NE.

**Methods:**

International databases with laser- and NE-related keywords were searched, and only randomized controlled trials (RCTs) that used any type of LLLT to treat NE and compared it with any type of control intervention were included. Eleven studies using laser acupuncture therapy (LAT), involving 927 participants, were included for a systematic review. A meta-analysis was conducted using full and partial response-rate variables. The analysis was performed using *P*referred *R*eporting *I*tems for *S*ystematic reviews and *M*eta-*A*nalyses guidelines, and the Cochrane risk-of-bias tool and *G*rading of *R*ecommendations *A*ssessment *D*evelopment and *E*valuation recommendations for quality of evidence were used to rate all included publications.

**Results:**

The LAT groups showed significant improvement, compared with control groups when full response rates were analyzed. There was no significant difference between the groups treated with LAT and the groups who underwent medication therapy alone when full response rates were analyzed. Red and infrared wavelengths and continuous waves were the most commonly used LAT modalities, and lower abdomen and back acupoints were the most-common sites.

**Conclusions:**

LAT seems to be an effective and safe treatment for NE; however, the quality of evidence available in the literature was relatively low. More-rigorous and higher-quality trials are needed to investigate this treatment modality further.

## INTRODUCTION

Nocturnal enuresis (NE), commonly known as bedwetting, is described as involuntary emptying of the bladder during sleep time in children older than 5 years.^1^ According to the International Children's Continence Society (ICCS), the condition is defined as primary monosymptomatic enuresis when a child has incontinence only at night without any other lower urinary-tract symptoms.^2^ The prevalence of NE can vary according to different regions and countries; a report from the United States showed a prevalence of 18% among school-age boys and 12% among school-age girls, as opposed to 8%–12% in Taiwanese children.^3,4^

Acupuncture, part of Traditional Chinese Medicine (TCM) treatment modalities, has been practiced for more than 2500 years in China,^5^ and acupuncture's popularity has increased among Western countries in recent decades and become a popular complementary medical treatment.^6,7^ In 2002, the World Health Organization (WHO) published a review on the efficacy of acupuncture in randomized controlled trials (RCTs) and outlined myriad medical conditions, as well as several urogenital conditions, for which acupuncture has been shown to be an effective treatment.^8^ In Asia, acupuncture has a long history as a primary therapy for NE, and evidence from clinical trials and systematic reviews of publications have shown that acupuncture has positive effects in NE management.^9–11^

The reputation of acupuncture as an effective treatment model, along with its safety and minimal side-effects, has fostered rapid growth in the development of other forms of acupuncture. Laser acupuncture therapy (LAT) is defined as photonic stimulation of acupuncture points and areas to initiate therapeutic effects similar to needle acupuncture therapies but with the benefits of low-level laser therapy (LLLT).^12^ Laser therapy or photobiomodulation is a treatment that uses photons at low-intensity, nonthermal laser irradiation to stimulate biologic activity in the body. LAT was first used in clinical practice in the 1970s,^13^ and records from China and Russia have reported the use of laser stimulation on acupuncture points, mainly for anesthesia purposes.^14^

The use of LAT for NE management has been suggested for clinical settings in both randomized and nonrandomized studies^15–17^ aimed at investigating the efficacy of LAT as an antienuretic therapy. Several systematic reviews of the use of acupuncture, LAT, and other complementary methods for NE treatment, have shown positive results, but methodological errors in the studies have resulted in low-quality evidence.^9,10,18,19^ In the most-recent systematic review published in 2015,^11^ the authors included several LAT publications; however, Chinese-language publications were not included, and more up-to-date reviewing that focused only on the use of LAT in NE management was needed. Therefore, there was a need for a more up-to-date review with an emphasis on LAT applications and laser parameters. Thus, the aim of this review was to evaluate the efficacy of LAT for treating pediatric NE through a systematic-review process.

## METHODS

### Search Strategy

Databases—Web of Science (*n* = 22), PubMed^®^ (*n* = 16), EMBASE^®^ (*n* = 16), the Cochrane Library (*n* = 18), and the China National Knowledge Infrastructure (CNKI; *n* = 45)—were searched for literature from 1971 to December 2020 and restricted the languages to Chinese and English. The following search key words were used in the English databases: enuresis OR nocturnal enuresis OR bedwetting AND laser OR laser therapy. For the CNKI database, the following search terms were used: Ji Guang AND Xiao Er Yi Niao. Two reviewers screened the articles independently according to their titles and abstracts and assessed them for relevance. This systematic review followed the *P*referred *R*eporting *I*tems for *S*ystematic reviews and *M*eta-*A*nalyses (PRISMA) criteria.^20^ See Supplementary Table S1 for the PRISMA checklist (supplementary data are available online at www.liebertonline.com/ACU).

The protocol of this systematic review was registered on PROSPERO, the international prospective register of systematic reviews (ID #: CRD42021225233).

### Inclusion/Exclusion Criteria

The review included articles based on the following inclusion criteria: (1) patients diagnosed with NE, either male or female, between 5–18 years old; (2) RCTs in English or Chinese; (3) experimental groups that used any type of LLLT applications for treatment of NE as a monotherapy or in conjunction with lifestyle recommendations, such as drinking habits and diet; (4) studies about LLLT interventions with any type of wavelength, either continuous or pulsed-wave, and an output power between 5 and 500 m*W* per second; (5) control group interventions of any type, such as sham LLLT and/or pharmacotherapy and/or acupuncture and/or Chinese herbal medicine; (6) studies of either acute or chronic NE stages; and (7) outcomes measured by response rates and partial response rates after treatment and/or after follow-up. The review excluded the following types of articles: (1) animal experiments, reviews, case reports, or letters to editor; (2) non-RCTs; (3) studies that included only forms of treatment other than LLLT applications, including pharmacotherapy, alarm treatment, or any type of Chinese Medicine (CM) treatment modality.

### Data Collection and Quality Assessment

Two reviewers (G.T. and C-H.L.) screened all articles independently according to the selection criteria, and potentially relevant articles were assessed further after the full texts of the articles were obtained. A standard form was used to extract the data and categorize it according to year of publication, author name and country, study design, sample size, mean age of each group's patients, type of intervention, number of sessions, follow-up time, acupoints used, and technical properties of laser application.

The Cochrane risk-of-bias tool^21^ was used for quality assessment of all included RCTs and 3 possible outcomes: (1) high; (2) unknown; or (3) low risk of bias.

The following criteria were used to assess the quality of each study: random sequence generation; allocation concealment; blinding of participants; blinding of study personnel; blinding of outcome assessor; completeness of outcome data; selective reporting; or other sources of bias (including insufficient reporting, unclear measurement assessment tools, or carryover effects in crossover trials). Two reviewers (G.T. and C-H.L.) performed the quality assessments, and any disagreement between the 2 reviewers was resolved by a discussion with a third author (Y-C.L.), whose opinion was used to resolve the disagreement. A standard form was used to extract the information for each study, which included the following characteristics: year of publication; name of author; country; type of study design; sample size; patients' mean age; type of intervention; control-group interventions; durations of treatment and follow-ups; acupoints used in the study; and technical parameters of laser application.

### Statistical Analysis

A meta-analysis was performed with Review Manager (RevMan) software, version 5.4 (The Cochrane Collaboration, 2020) using full response rate and partial response rate measurements. Comparisons between laser and pharmacotherapy interventions and with a follow-up of 6 months were analyzed. Continuous data were shown as mean difference (MD) ± standard deviation (SD) with a 95% confidence interval (CI), and a *P*-value <0.05 was considered significant. Heterogeneity was assessed between the groups using the χ^2^ and *I^2^* tests. In cases of heterogeneity higher than 50%, a random-effect model was used and, if the *I^2^* tests were below 50%, a fixed-effect model was used for analysis estimation. Quality of evidence analysis was conducted based on the *G*rading of *R*ecommendations *A*ssessment *D*evelopment and *E*valuation (GRADE) guidelines.^22^ Each Forest-plot estimation was graded with the GRADEpro GDT online system, using the following criteria: risk of bias; inconsistency; indirectness; imprecision; and other considerations. GRADE specifications were categorized as high, moderate–low, and very low.

## RESULTS

**FIG. 1.**
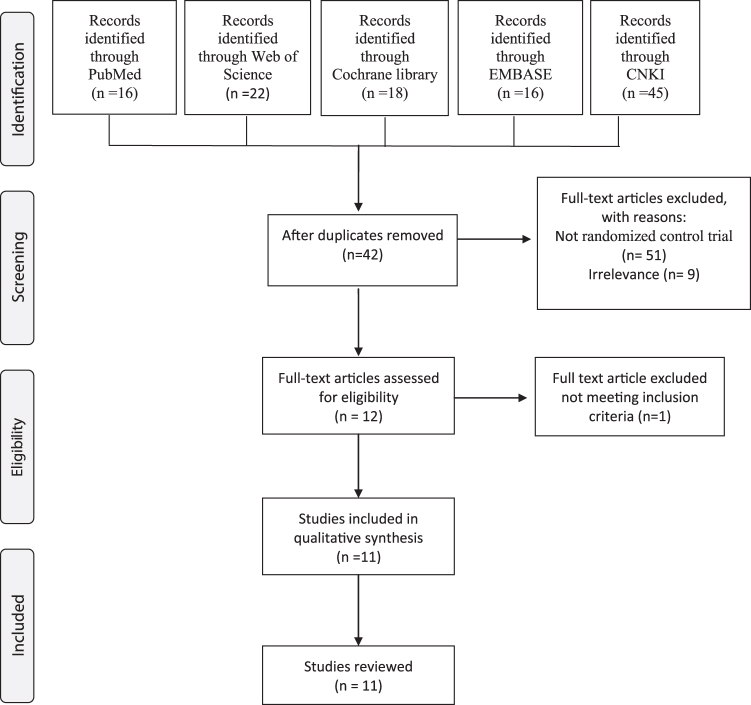
Study flowchart depicting the rationale for the selected studies and follows the *P*referred *R*eporting *I*tems for *S*ystematic reviews and *M*eta-*A*nalyses (PRISMA) statement. CNKI, China National Knowledge Infrastructure.

A total of 117 studies were identified based on the search key words in the selected databases. After an initial review of all abstracts, 42 duplicates, 51 non-RCTs, and 12 irrelevant articles were removed, resulting in 12 full-text articles that were assessed critically for eligibility, based on the inclusion/exclusion criteria. Finally, after 1 article was excluded due to the use of both LLLT and medication in the intervention group, 11 relevant articles were included and reviewed by 2 independent researchers (G.T. and C-H.L.; Fig. 1).

### Characteristics of Selected Studies

Table 1 shows the characteristics of the included studies. Eleven studies from the years 1995 to 2016 were included; 6 were in English^23–28^ and 5 were in Chinese.^29–33^ A total of 927 NE participants, which included 512 in the LAT groups and 415 in the control groups, were analyzed. Two studies did not report gender differences,^25,28^ which resulted in 535 (58%) male participants and 384 (42%) female participants in the remaining studies. The mean number of participants for each study was 84, and the mean age was 7.47 years. Of note, 1 study of resistant monosymptomatic NE included 15-year-old patients.^26^ Three studies were designed as double-blinded RCTs^23–25^; 3 were parallel, 3-armed studies with 2 control groups, including 2 studies that combined LLLT therapy and pharmacotherapy^26,28^; and 1 study used a placebo laser treatment with and without contact.^24^ The remaining studies were parallel and 2-armed with 1 control group, which included medications, placebo LLLT, body and auricular acupuncture, and Chinese herbal medicine. None of the included studies reported the use of *St*andards for *R*eporting *I*nterventions in *C*linical *T*rials of *A*cupuncture.

**Table 1.** Characteristics of Included Studies in the Systematic Review

Study #	1****st**** author, yr and ref	Country	Design	Sample size IG	Sample size CG	Age IG	Age CG	Type of Intervention IG	Type of Intervention CG	Duration of treatment (wks)	# of total sessions/frequency (S/wk)	Follow-up (mos)
1	Alsharnoubi, 2017^28^	Egypt	RCT	15	15, 15	8.8 ± 3.18	9.43 ± 2.77	LAT	1.Desmopressin 2. LAT+ desmopressin	12	24 (2/12)	6
2	Gong, 2008^32^	China	RCT	102	62	10	NM	LAT	Acupuncture	2	10	3
3	Karaman, 2011^23^	Turkey	Double-blinded RCT	57	26	8.5	NM	LAT	Placebo LAT	4	12 (3/4)	6
4	Lee, 2004^29^	China	RCT	56	50	6.27	NM	LAT	Imipramine	2–3	10–20 (NM)	NM
5	Mogahed, 2016^25^	Egypt	Double-blinded RCT	25	25	10.84 ± 3.31	10.28 ± 3.27	LAT	Placebo LAT	4	12 (3/4)	0
6	Moursy, 2014^26^	Egypt	RCT	62	62, 62	15.6	15.9	LAT	1. Desmopressin 2. LAT + desmopressin	12	24 (2/12)	6
7	Radmayr, 2001^27^	Austria	RCT	20	20	8	8.6	LAT	Desmopressin	4–5	12.45 (3/4–5)	6
8	Radvanska, 2011^24^	Slovakia	Double-blinded RCT	16	13	8.5 ± 3.2	8.9 ± 3.3	LAT	1. Placebo LAT contact 2. Placebo LAT no contact	5	12 (2/3 & 3/2)	0
9	Yuan, 1995^33^	China	RCT	32	30	5.77	NM	LAT	CHM	NM	NM	NM
10	Zhu, 1999^30^	China	RCT	87	72	7.67	NM	LAT	CHM + AT	4	10 (3/3)	6
11	Zhuang, 2004^31^	China	RCT	40	40	7.5	NM	LAT	Imipramine, chlorhexidine	2–4	10–30	NM

yr, year; IG, intervention group; CG, control group; wk(s), week(s); S, session; mos, months; RCT, randomized control trial; LAT, laser acupuncture therapy; NM, not mentioned.; CHM, Chinese herbal medicine; AT, auricular therapy.

### Quality of Selected Studies

**FIG. 2.**
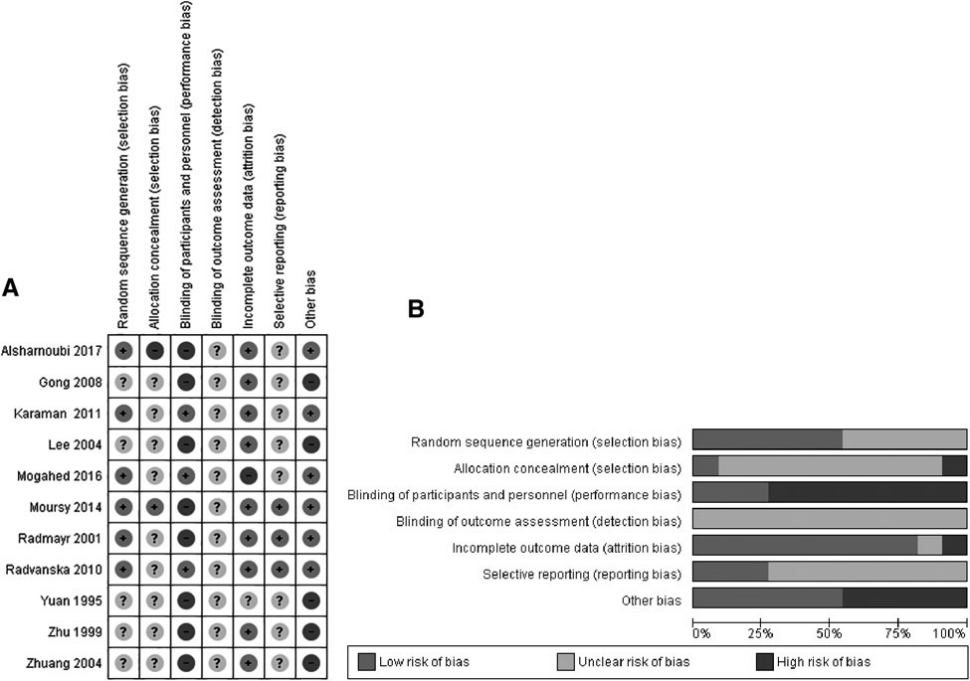
Risk of bias of all included studies. **(A)** Risk-of-bias summary **(B)** Risk-of-bias graph.

Figure 2 shows the quality assessment of the included RCTs using the Cochrane risk-of-bias tool. Figure 2A shows the risk-of-bias summary, which includes the reviewers' judgments about each risk-of-bias item for all included studies. Figure 2B shows the risk-of-bias graph, which includes the reviewers' judgments about each risk-of-bias item, presented as percentages across all included studies. In the criteria of random-sequence generation, 5 studies did not report randomization clearly and were rated with an unclear risk of bias.^29–33^ In terms of allocation concealment, only 1 study^26^ clearly reported the concealment method, and none of the remaining studies reported it at all. Only 3 double-blinded studies blinded their participants properly,^23–25^ while the rest of the studies did not report blinding or could not blind the participants or personnel due to study design. None of the studies reported clearly whether outcome assessment was blinded or not. The majority of studies reported complete outcome data but 2 studies provided incomplete data.^25,33^

Five studies^29–33^ did not report adverse effects (AEs) clearly, and 3 studies,^23,25,28^ deliberately or not, did not report AEs accurately, and, therefore, were rated with an unclear risk of bias. Finally, with regard to other sources of bias, 5 studies^29–33^ were rated with a high risk of bias due to unclear measurement tools and overall reporting.

GRADE guidelines were used to assess evidence quality and several studies^29–33^ were strongly suspected of publication bias and obfuscation.

### Laser Parameters and Acupoints in the Selected Studies

**FIG. 3.**
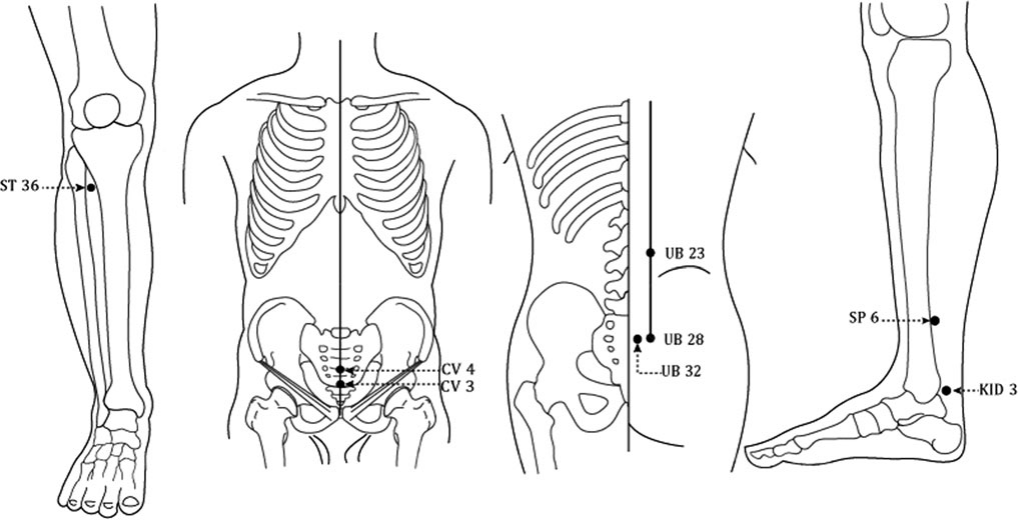
Most commonly used acupoints in the included studies: ST 36, CV 3, CV 4, UB 23, UB 28, UB 32, SP 6, and KID 3.

Table 2 shows the technical properties and types of laser devices used for NE treatment. Most of the studies applied either red (632–670 nm) or infrared (808–905 nm) wavelength lasers and used a laser-pen device to stimulate acupoints in the lower abdomen, lumbar, sacrum, and lower limbs (Fig. 3). Only 2 studies^28,30^ used pulsed-wave lasers, while the rest of the studies used continuous-wave lasers. The most commonly used acupoints were *San Yin Jiao* (SP 6), *Guan Yuan* (CV 4), *Zhong Ji* (CV 3), *Shen Shu* (UB 23), *Pang Guang Shu* (UB 28), *Zu San Li* (ST 36), *Ci Liao* (UB 32), and *Tai Xi* (KID 3). See Table 3.

**Table 2.** Technical Properties of Laser Used in Included Randomized controlled Trials

Study#	1st author, yr &ref	Type of laser device	Wavelength (nm)	Frequency (Hz)	Time in sec (each point/# of points/total time)	Power m*W*/energy density J/cm^2^/total energy
1	Alsharnoubi, 2017^28^	Giotto Med laser machine	905	2500hz	60/11/660	1500/NM/NM
**2**	Gong, 2008^32^	Helium–Neon laser pen, type JG-I	632	NM	300/5–9/1500–2700	4/1.2/6–10.8
**3**	Karaman, 2011^23^	Gallium arsenide (GaAs)	635-670	NM	60/7/420	5/0.3/2.1
**4**	Lee, 2004^29^	Helium-Neon laser pen	NM	NM	600/5/3000	15/09/27
**5**	Mogahed, 2016^25^	Helium-Neon laser pen	632	NM	60/15/900	15/0.6/9
**6**	Moursy, 2014^26^	Gallium aluminum arsenide (GaAlAs) laserpen	808	NM	26/10/260	200/4/40
**7**	Radmayr, 2001^27^	NM	670	NM	30/7/210	10/0.3/2.1
**8**	Radvanska, 2011^24^	Gallium arsenide (GaAs) laser pen	670	NM	20/16/320	NM
**9**	Yuan, 1995^33^	Helium-Neon laser pen, type LJ-3	632	NM	180/NM/NM	NM
**10**	Zhu, 1999^30^	Helium-Neon laser pen, type 8-4	632	50hz	300/10/3000	16/2.4/24
**11**	Zhuang, 2004^31^	Helium-Neon laser pen, type J.2Y-450	632	NM	1200/5/NM	3/NM/NM

yr, year; sec, seconds; J/cm^2^, Joules per square centimeter; NM, not mentioned.

**Table 3.** Acupuncture Points^a^ in Included Studies

Study	CV 2	CV 3	CV 4	CV 6	CV 12	DU 4	DU 20	ST 29	ST 36	SP 6	HT 7	UB 18	UB 20	UB 23	UB 28	UB 32	UB 40	UB 58	KD 3	LV 3	Yi Niao
1	×	×	×							×				×	×	×					
2		×	×	×	×	×	×		×	×				×	×						
3		×	×	×					×	×											
4		×								×											×
5		×	×	×	×				×	×			×	×	×	×			×		
6	×	×	×					×	×	×	×			×	×	×	×				
7			×				×		×	×				×					×		
8		×	×			×			×	×	×			×					×	×	
9			×							×		×	×	×				×	×		
10		×	×							×				×	×						×
11		×	×							×				×		×					

^a^ World Health Organisation alphanumeric code of acupuncture points is used in this table.

### Outcome Measures and Results

The main outcome measure in all included studies was response rate, which was defined according to the ICCS standard definition of lower urinary-tract dysfunction in children. Full response was defined as a minimum of 90% reduction in the number of wet nights; partial response was a minimum of 50% reduction in the number of wet nights, and nonresponse was characterized as less than a 50% reduction in the number of wet nights.

**FIG. 4.**
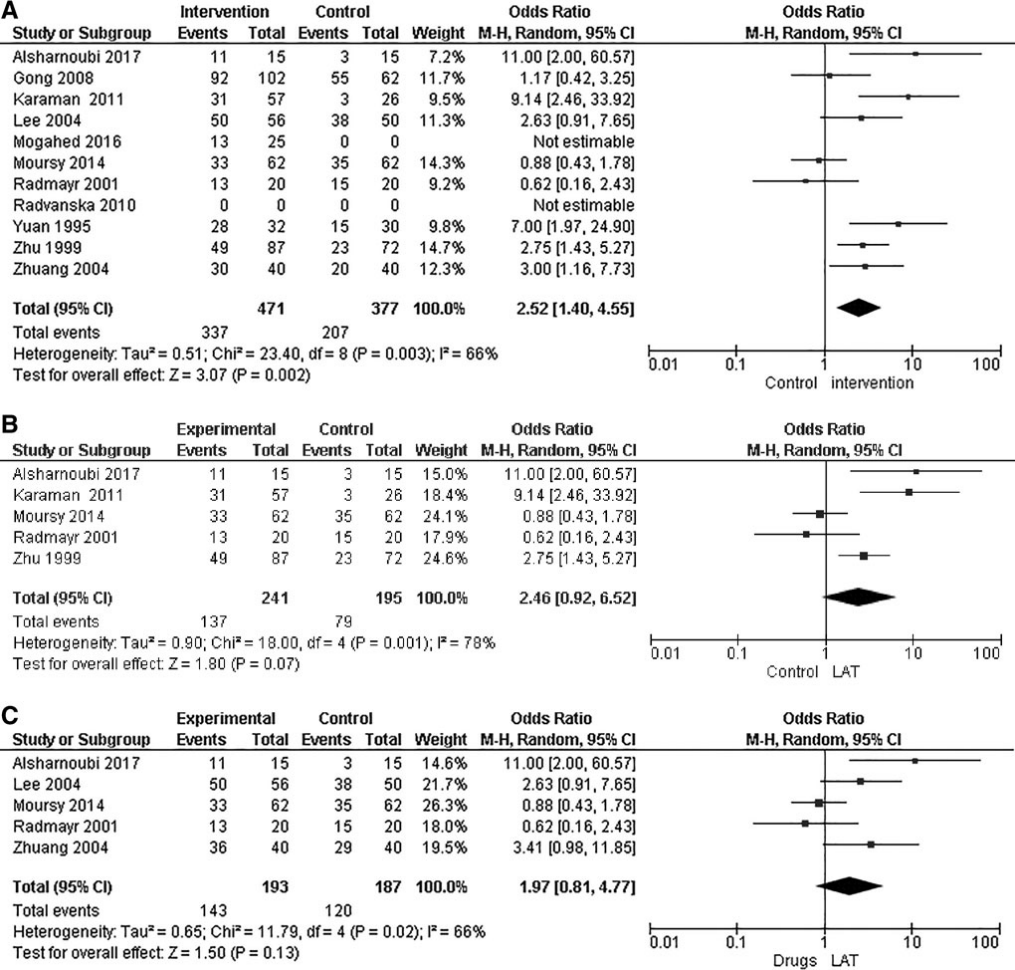
**(A)** Forest plot of full response rate of laser acupuncture therapy (LAT) group versus all types of control groups. Forest plot of comparison included only LAT versus control groups that included sham LAT, medication therapy, acupuncture, and Chinese herbal medicine + auricular acupuncture. Follow-up times for each of the 11 studies were: (1) 6 months; (2) 3 months; (3) 6 months; (4) NM; (5) 1, (6) 6 months, (7) 6 months; (8) 1 month, (9) NM, (10) 6, (11) NM. **(B)** Forest plot of full response rate of LAT group versus control group in a follow-up of more than 6 months. Forest plot of comparison included LAT versus control groups, which included: sham LAT; medication therapy; LAT with medication therapy; and Chinese herbal medicine combined with auricular acupuncture. **(C)** Forest plot of full-response rate of LAT group versus a control group of pharmacotherapy. Forest plot of comparison included only LAT versus control groups that included the following pharmacotherapies: desmopressin; imipramine; and chlorhexidine. Follow-up times for study numbers 1, 4, 6, 7, and 8 were: (1) 6 months; (4) NM; (6) 6 months; (7) 6 months; (8) 0 months. NM, not mentioned; M-H, Mantel–Haenszel; CI, confidence interval.

A meta-analysis was conducted, and 9 of the 11 studies showed significant full-response rates when patients were treated with LAT, compared with control groups for participants with follow-up times between 1 and 6 months (Mantel–Haenszel [M-H]: 2.52; 95% CI: 1.40, 4.55; *P* = 0.002; *I^2^* = 66%; Fig 4A).

Two studies were not included in the analysis: 1 study^25^ had incomplete data and did not respond to the current authors' contact; and 1 study^24^ reported no statistically significant full response rate.

For studies with a 6-month follow-up, meta-analysis of 5 of them showed a borderline significance in full response rate when patients were treated with LAT, compared with control interventions (M-H: 2.46; 95% CI: 0.92, 6.52; *P* = 0.07; *I^2^* = 78%; Fig. 4B).

When comparing full response rates to treatment with LAT versus pharmacotherapy alone (with desmopressin, imipramine, or chlorhexidine), a meta-analysis of 5 studies showed no significant difference in full-response rates at follow-ups between 1 and 6 months (M-H: 1.97; 95% CI: 0.81, 4.77; *P* = 0.13; *I^2^* = 66%; Fig. 4C). In this Forest plot, 2 studies had a third group of treatment that combined LAT and pharmacotherapy, and thus, were not included in the analysis.^26,28^

**FIG. 5.**
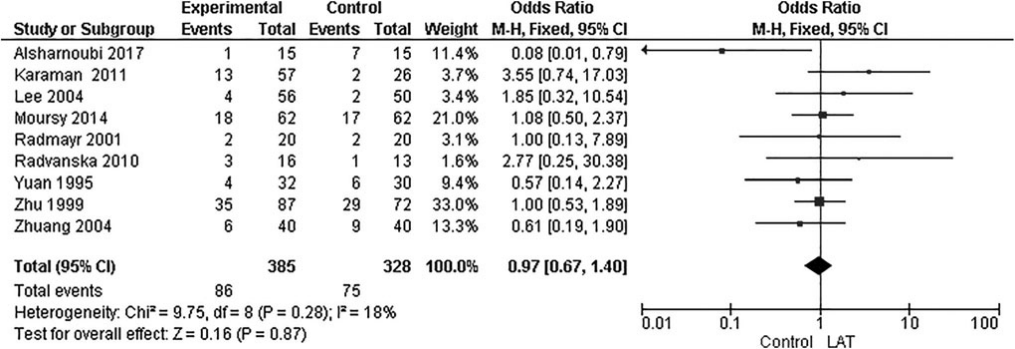
Forest plot of partial response rate of laser acupuncture therapy (LAT) group versus control group. Forest plot of comparison included LAT versus control groups, which included: sham LAT; medication therapy; LAT with medication therapy; and Chinese herbal medicine with/without auricular therapy. M-H, Mantel–Haenszel; CI, confidence interval.

In a comparison of partial response rates of groups treated with LAT versus control groups, a meta-analysis of 9 studies showed no significant differences between the LAT groups and the control groups at follow-up times of 1–6 months (MD: 0.97; 95% CI: 0.67, 1.40; *P* = 0.87; *I^2^* = 18%; Fig. 5). In addition to the studies that compared groups treated with LAT and control groups treated with medication alone, (Fig. 4C), there were several types of control groups among the studies that used sham LAT, medication therapy, LAT with medication therapy, body and auricular acupuncture, and Chinese herbal medicine with auricular acupuncture.

In terms of weekly bedwetting frequency, 3 studies^23,26,28^ reported a significant reduction in bedwetting when patients were treated with LAT, compared with control groups during a follow-up of 6 months, while 1 study^24^ reported no significant difference between a group receiving LAT treatment and a control group. In terms of maximum voided volume (MVV), 2 studies^25,26^ reported a positive effect on voiding frequency during daytime in groups treated with LAT, compared with control groups during a follow-up of 6 months, while 1 study^24^ reported no effect after treatment in any of the groups. None of the included studies used expectancy and belief measures as part of their outcome measurements. See Supplementary Table S2 for a summary of the evidence. 

## DISCUSSION

NE is a distressing and stressful medical condition that causes significant psychologic distress to enuretic children and their families. NE is also associated with increased risks of social, emotional, and behavioral comorbidities.^34^ Although pharmacotherapy is considered to have a short-term effect among children with enuresis,^35^ parents are often hesitant to initiate pharmacologic treatments.^36^ Therefore, novel complementary approaches to treating children with NE are still needed.

### Summary of the Evidence

This systematic review was the first investigation to evaluate the efficacy of LAT in clinical trials for treating pediatric NE. The data analyzed included 11 RCTs involving 927 participants, and all included studies used LAT in the experimental groups versus pharmacotherapy, placebo laser, or different CM treatment modalities (such as acupuncture or Chinese herbal medicine). Based on results of the analysis, LAT shows clinical efficacy for treating children with enuresis and is an attractive option for parents because it is a safe, noninvasive pain-free method and is easy to administer.

Three double-blinded studies^23–25^ compared LAT with sham LAT. Karaman et al.^23^ enrolled 91 participants, applied red light (635–670-nm wavelength) for 4 weeks, and reported that the full-response rate for LAT was significantly higher (M-H: 9.14; 95% CI: 2.46, 33.92; Fig. 4A). The researchers for this study reported that application of LAT produced significantly decreased mean numbers of bedwetting incidents and a better partial response after analysis of weekly bedwetting, compared with placebo LAT.^23^ Mogahed et al. studied 50 participants, applied red light (632-nm wavelength) for 4 weeks, and reported that LAT was significantly more effective for decreasing the mean number of weekly bedwetting incidents and improving bladder-reservoir function, compared with placebo LAT.^25^ In contrast, Radvanska et al. studied 29 participants, applied red light (670-nm wavelength) for 5 weeks, and reported that LAT treatment was not significantly better than placebo LAT^24^; however, the real laser treatment did produce a significant improvement in average voided volume during the day, which indicated some effect on bladder-reservoir function.

Five studies compared LAT treatment with pharmacotherapy.

Alsharnoubi et al.^28^ studied 45 participants in 3 groups, comparing treatment with LAT only, desmopressin only, and a combined treatment with both LAT and desmopressin. Infrared light (905-nm wavelength) was applied for 12 weeks, and it was reported that treatment with LAT had a significantly higher response rate, compared with the other 2 groups (M-H: 11; 95% CI: 2.00, 60.57; Fig. 4C). Desmopressin was administered sublingually once per day for 3 months at a dose of 0.06 mg. The researchers reported that the LAT group showed significantly decreased bedwetting frequency, increased bladder capacity, and produces the overall lowest relapse rate (not statistically significant), compared with the other 2 groups.

Lee et al.^29^ studied 106 participants who underwent treatment with LAT for 2–3 weeks, and Lee et al. reported that the LAT group had a significantly higher response rate, compared with a group treated with imipramine (M-H: 2.63; 95% CI: 0.91, 7.65; Fig. 4C). Imipramine was administered orally once per day for 14 days in a dose of 12.5–25 mg.

Moursy et al.^26^ studied 186 participants with resistant monosymptomatic NE, who were divided into 3 groups that compared LAT only, desmopressin only, and a combined treatment. LAT (808-nm wavelength) was applied for 12 weeks, and no significant differences between the groups treated with LAT and those treated with desmopressin monotherapy were reported (M-H: 0.88; 95% CI: 0.43, 1.78; Fig. 4C). However, the combined treatment group had a significantly higher response rate, compared with the 2 monotherapy groups. Desmopressin was administered once per day for 3 months at a lyophilisate sublingual dose (MELT) of 120 g. The researchers reported that the MVV of all 3 groups was significantly better at 6 months than at baseline.

Radmayr et al.^27^ studied 40 participants and applied red light (670-nm wavelength) for 5 weeks and reported no significant differences between a group treated with LAT and a group treated with desmopressin (M-H: 0.62; 95% CI: 0.16, 2.43; Fig. 4C). Desmopressin was administrated once per day for 3 months intranasally at a dose of 0.02–0.04 mg.

Zhuang et al.^31^ studied 80 participants and applied red light (632-nm wavelength) for 2–4 weeks and reported that the group treated with LAT had a significantly higher full response rate, compared with groups treated with imipramine and chlorhexidine (M-H: 3.41; 95% CI: 0.98, 11.85; Fig. 4 C). Imipramine and chlorhexidine medications were administered once per day for 1 month. The researchers did not report the dose of the medications.

Three studies compared treatment with LAT versus treatment with either body and auricular acupuncture, Chinese herbal medicine, or a combination of both. Gong et al.^32^ studied 164 participants and applied red light (632-nm wavelength) for 2 weeks. The researchers reported that a group treated with LAT had an overall higher full response rate, compared with an acupuncture group (M-H: 1.13; 95% CI: 0.58, 2.18; Fig. 4A). Yuan et al.^33^ studied 62 participants and reported that a group treated with LAT had a significantly higher full response rate, compared with a group treated with Chinese herbal medicine (M-H: 7.00; 95% CI: 1.97, 24.90; Fig. 4A). Zhu et al.^30^ studied 152 participants, applied red light (632-nm wavelength) for 4 weeks, and reported that a group treated with LAT had a significantly higher full response rate, compared with a group treated with Chinese herbal medicine and auricular therapy (MD: 2.75; 95% CI: 1.43, 5.27: Fig. 4A).

### Laser Acupuncture Therapy and Photobiomodulation

Current evidence shows that laser irradiation with either red or infrared light on acupoints located mainly in the lower abdomen and lumbar areas can increase nocturnal bladder capacity, decrease the weekly number of wet nights, and improve the overall recovery rate in children with enuresis. While other wavelengths—such as violet, blue, green, and yellow—have a shallow penetration in human tissue, compared to red light, the nonvisible infrared light had the same penetration depth as the red light,^13^ and, therefore, these 2 types of wavelength are most commonly used.

Previous investigations of acupuncture use also discovered similar findings of increased nocturnal bladder capacity^9,37^ and speculated an antidiuretic mechanism. While these studies produced a better response when acupuncture was combined with another therapy, the majority of included studies in this systematic review showed either a superior or equal effect of LAT, compared with either pharmacotherapy or sham controls. With regard to the possible placebo effect of either acupuncture or LAT that can last for 4 weeks, 6 of the 11 included studies mentioned long-term follow-ups, while 3 studies did not mention a follow-up and 2 studies did not follow the patients after the treatment (Table 1).

According to the theory of TCM, a holistic system of channels and organs exists in the body, and stimulation of acupoints along these channels corrects Disharmony and dysregulation of organ systems.^36^ Children with enuresis benefit from the therapeutic effects of acupoints to relieve their symptoms and from photonic stimulation and LLLT, which may influence the spinal micturition centers and parasympathetic innervation of the urinary-tract system.^27^ Thus, it seems that LAT, like acupuncture, has the effect of suppressing spinal and supraspinal reflexes and increasing ß-endorphin levels, which can inhibit bladder contractions.^11^

### Limitations

This systematic review had notable limitations. First, according to the Cochrane risk-of-bias tool, methodological bias existed in all the included studies, and in a few studies, there was a high risk of bias (Fig. 2). There was 1 study^28^ that could not be evaluated because the allocation was concealed, but none of the other studies had adequate allocations and did not report their randomization procedures clearly. Blinding was lacking in all of the studies, and even the double-blinded studies^23–25^ did not report clearly how the administering therapist or the assessors were blinded. The rest of the studies did not report any type of blinding, including the participants, the therapists, or the assessors. A high risk of selection bias existed in 1 study,^28^ and it seemed that nonmonosymptomatic NE participants, who generally have more severe and serious enuresis symptoms, were unclassified and possibly enrolled in 1 of the control groups.^38^

Quality of evidence according to GRADE guidelines was also very low in all meta-analysis plots due to publication bias and indirectness. Some studies lacked proper explanations and seemed to have missing data, including participants' baseline characteristics and laser parameters. Several attempts were made (unsuccessfully) to contact the researchers involved with the study done by Mogahed et al.^25^ to obtain their missing data, and none of the Chinese publications included contact information for discovering the laser parameters.

The follow-up times for the majority of included studies was at least 3 months, and 5 studies even had 6-month follow-up times, which is considered a standard time to assess response and relapse rates in EN.^35^

The primary outcome in all included studies was response rate; therefore, the current meta-analysis focused on this outcome measurement. However, more outcome measures are recommended to assess the effectiveness of LAT for treating NE, including relapse rate, bladder capacity, MVV, and daytime and nocturnal urine production. Future double-blinded sham-controlled studies should investigate the efficacy of LAT as a monotherapy or as part of a treatment combined with medications, compared with sham treatment.

## CONCLUSIONS

The current findings suggest that using LAT may have clinical effects on NE; however, the quality of evidence was relatively low, and further rigorous high-quality trials are still needed to support the use of LAT in NE. The results of this review should be taken into account cautiously due to the heterogeneity of the included studies. Nevertheless, LAT is a safe, noninvasive, pain-free, and fast method that is easy for the physician to apply and easy for the pediatric patient to undergo. These characteristics and the current available evidence justify further large-scale studies on this topic.

## AUTHORS' CONTRIBUTIONS

Drs. Ton, Yen, and Lee conceptualized the study, and together with Dr. Lai, were responsible for the study design. Dr. Lee was the principal investigator. Drs. Ton and Lin performed data collection and analysis, while Dr. Ho performed the statistical analysis. Dr. Ton drafted the article, and all of the authors approved its final version.

## Supplementary Material

Supplemental data

Supplemental data
