# Supplementary material for: The Effects of Laser Acupuncture Therapy on Nocturnal Enuresis: A Systematic Review and Meta-Analysis
Source: Med Acupunct. 2022 Aug 17;34(4):228–39. doi: 10.1089/acu.2022.0002 (PMC9419947; doi:10.1089/acu.2022.0002)
Supplement: Supplemental data [file Suppl_TableS2.pdf]

Summary of findings:

LAT compared to Control group for Nocturnal enuresis

Patient or population: Nocturnal enuresis
Setting:
Intervention: LAT
Comparison: Control group

Outcomes	Anticipated absolute effects* (95% CI)		Relative effect (95% CI)	№ of participants (studies)	Certainty of the evidence (GRADE)	Comments
	Risk with Control group	Risk with LAT				
LAT vs control	507 per 1,000	**716 per 1,000** (585 to 818)	**OR 2.45** (1.37 to 4.38)	848 (11 RCTs)	⨁◯◯◯ VERY LOW a,b,c,d	
LAT vs medication	642 per 1,000	**779 per 1,000** (592 to 895)	**OR 1.97** (0.81 to 4.77)	380 (5 RCTs)	⨁◯◯◯ VERY LOW a,b,c,d	
LAT vs control follow up: 6 months	405 per 1,000	**671 per 1,000** (484 to 815)	**OR 2.99** (1.38 to 6.45)	436 (5 RCTs)	⨁◯◯◯ VERY LOW a,b,c,d	
LAT vs control (partial response)	229 per 1,000	**223 per 1,000** (166 to 293)	**OR 0.97** (0.67 to 1.40)	713 (9 RCTs)	⨁◯◯◯ VERY LOW a,b,c,d	

***The risk in the intervention group** (and its 95% confidence interval) is based on the assumed risk in the comparison group and the **relative effect** of the intervention (and its 95% CI).

CI: Confidence interval; **OR:** Odds ratio

GRADE Working Group grades of evidence
High certainty: We are very confident that the true effect lies close to that of the estimate of the effect
Moderate certainty: We are moderately confident in the effect estimate: The true effect is likely to be close to the estimate of the effect, but there is a possibility that it is substantially different
Low certainty: Our confidence in the effect estimate is limited: The true effect may be substantially different from the estimate of the effect
Very low certainty: We have very little confidence in the effect estimate: The true effect is likely to be substantially different from the estimate of effect

Explanations

a. some concern about with allocation concealed and blinding for therapists who administreted the therapy and assessors who measured at least one key outcome.
b. some concern about the heterogeneity (more than 50%)
c. High concern about the difference and variation between intervention groups and control group
d. imprecise confidence interval
